# Supplementary material for: Association between oral microbiome and seven types of cancers in East Asian population: a two-sample Mendelian randomization analysis
Source: Front Mol Biosci. 2023 Nov 21;10:1327893. doi: 10.3389/fmolb.2023.1327893 (PMC10702768; doi:10.3389/fmolb.2023.1327893)
Supplement: Supplementary file 1 [file DataSheet1.ZIP › Supplementary Materials/MR plots of saliva/saliva═╝/ovarian cancer/pheno.66_to_ovarian cancer_forest.pdf]

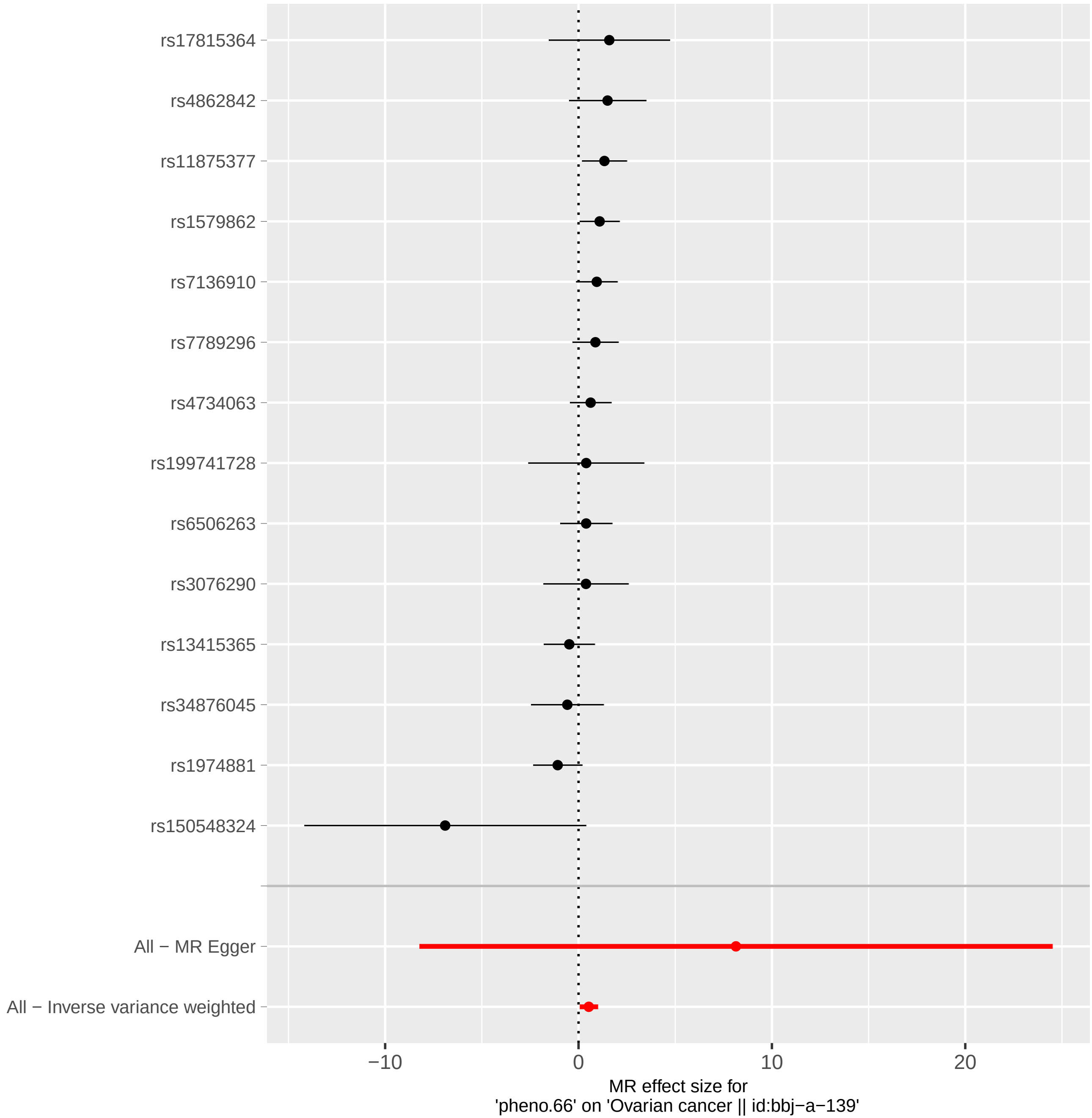
rs17815364
rs4862842
rs11875377
rs1579862
rs7136910
rs7789296
rs4734063
rs199741728
rs6506263
rs3076290
rs13415365
rs34876045
rs1974881
rs150548324
All − MR Egger
All − Inverse variance weighted
−10
0
10
20
MR effect size for
'pheno.66' on 'Ovarian cancer || id:bbj−a−139'
